# Effect of inhaled anaesthetics gases on cytokines and oxidative stress alterations for the staff health status in hospitals

**DOI:** 10.1007/s00420-021-01705-y

**Published:** 2021-05-06

**Authors:** Khaled A. AL-Rasheedi, Abdulmajeed A. Alqasoumi, Ashraf M. Emara

**Affiliations:** 1 Khyber General Hospital, Ministry of Health, Khyber, Saudi Arabia; 2 Department of Pharmacy Practice, College of Pharmacy, Qassim University, Qassim, Saudi Arabia; 3 Department of Pharmacology and Toxicology, College of Pharmacy, Qassim University, Buraidah, Qassim Saudi Arabia; 4 Department of Clinical Toxicology, Faculty of Medicine, Tanta University, Tanta, Egypt

**Keywords:** Inhaled anaesthetics, Cytokines, Oxidative stress, Staff health, Hospitals

## Abstract

**Objectives:**

The present study aimed to evaluate the effects of waste anaesthetic gases on cytokines and oxidative stress of hospital health team members following exposure to waste anaesthetic gases (WAGs).

**Subjects and methods:**

In total, 180 participants took part in this study; 60 of these were healthy male controls and the 120 participants in the intervention group were staff who work in the operating room. This latter group comprises six occupational subgroups (1) surgeons, (2) surgical assistants, (3) anaesthesiologists (4) anaesthesiology assistants, (5) nurses and (6) janitors. The following parameters were assessed: catalase (CAT), glutathione peroxidase (GSHpx) and superoxide dismutase (SOD) activities, plasma fluoride, serum interferon gamma (IFN-γ), serum interleukin 2 (IL2), serum interleukin 4 (IL4) and plasma thiobarbituric acid reactive substances (TBARS).

**Results:**

Anaesthesiologists and their assistants exhibited the highest levels of plasma fluoride, serum IFN-γ and IL 2, exceeding the levels in detected in all the other occupational subgroups. Furthermore, the serum levels of IL4 were significantly raised in anaesthesiologists and the difference between this group and other groups was statistically significant. However, compared with the other subgroups, surgeons exhibited elevated plasma TBARS and reduced CAT, GSHpx and SOD; these variances were also statistically significant.

**Conclusion and recommendations:**

The findings of this study indicate that operating room staff exposed to WAGs are vulnerable to experiencing immunotoxicity as the WAGs are considered to initiate oxidative stress and increase the levels of cytokines in serum. Thus, an education programme is warranted to inform staff working in environments where they may be subjected to WAGs on the effects that the gases can have upon their health and how to minimise their exposure to WAGs. An ongoing effort is also needed to ensure anaesthesia safety standards are maintained at all times. The findings of this study may provide a springboard for future research into occupational exposure to WAGs and their wider effect upon health.

## Introduction

When anaesthetics are administered, small amounts of volatile waste anaesthetic gases (WAGs) and vapour escape from the patient's breathing apparatus into the operating room environment (Yasny and White 2012).

WAG pollution of operating room environment is attributed to three factors: the anaesthesia workstation, anaesthetic administration techniques and the availability of a scavenging system (Yasny 2012). However, WAGs occur for several reasons, including (1) gaseous anaesthesia being delivered using a facemask. (2) Leaving the gas valve flow meter and vaporiser switched on. (3) Anaesthetic spillage during charging of the vaporiser. (4) Undertaking flushing after finishing surgery to speed up recovery from inhaled anaesthesia (a practice that is both common and deleterious). (5) Issues with the facemask, such as being the wrong size for the patient or being made of unsuitable material; or the patient has an airway abnormality. (6) Imperfect laryngeal mask inflation or application of endotracheal tube cuff or the endotracheal tube is uncuffed, leading to anaesthesia gases being released into the environment (Lucio et al. 2018).

During the administration of inhalation anaesthetics, all staff in the operating room environment are exposed to the volatile compounds and are at risk of experiencing adverse effects to them (Tanko et al. 2014). The Saudi Arabian Ministry of Health states that the inhaled anaesthetics that were used most commonly in 2014 were isoflurane and sevoflurane. Owing to its airway and heart safety record, sevoflurane is the anaesthetic most frequently used for surgical patients in hospitals.

Aragonés et al. (2016) are among the many researchers who have documented that chronic exposure to high levels of inhaled anaesthetics has deleterious effects upon health. Fatigue, headache and irritability are among the symptoms that are associated with excessive exposure, which can also lead to cardiopulmonary and liver problems (Misa et al. 2016).

Oxidative stress is the imbalance between the production of free radicals and intrinsic antioxidant defences (Elgharabawy and Emara 2014; Aldubayan et al. 2019). Lim (2014) explained that the body maintains the balance between ROS and antioxidants through several enzymatic and non-enzymatic antioxidant systems. These systems and their protective influence against various chronic conditions have been the focus of research conducted by Emara et al. (2010). Chronic inflammatory responses are also significantly influenced by oxidative stress recruiting immune cells to sites of inflammation (El-Gharieb et al. 2010; Al-Rasheed et al. 2017). Paradoxically, chronic inflammation also escalates oxidative stress due to elevated quantities of inflammatory mediators in the area. Thus, balance is also required for inflamed responses; moderate acute inflammation serves the purpose of protecting the body against harmful pathogens; but if inflammation that arises from stress-induced cellular dysfunction becomes protracted, there is an increased risk that it will become a chronic condition (Wu et al. 2012). The inflammatory process is heightened by inflammatory cells secreting cytokines and other soluble mediators that release ROS, which attracts other cells to damaged tissue (Ferguson 2010).

Orhan et al. (2013) claimed that surgical patients who have received isoflurane and sevoflurane experience an increase in lipid and protein oxidation. The DNA of peripheral lymphocytes was damaged and their level glutathione was reduced in orthopaedic surgery patients given sevoflurane. As in glutathione is a ROS scavenger, its reduction has implications for the cell’s redox state (Franco and Cidlowsk 2012).

In the study conducted by Braz et al. (2020), young medical residents working in operating rooms that did not have adequate scavenging systems and thus were exposed to high concentrations of WAGs for three years, did not exhibit oxidative stress; however, genomic damage and inflammatory states were detected. Similarly, medical staff exposed to high concentrations of anaesthetic gases, even for short durations were found to have elevated levels of IL-8, a pro-inflammatory chemokine (Chaoul et al. 2015). Whilst many published papers report on the adverse effect of anaesthetics upon inflammation and oxidative stress in patients, the literature barely considers the effect of WAG exposure upon healthcare workers. Therefore, this study aims to categorise the effects upon the cytokines and oxidative stress of operation room staff who are chronically exposed to inhaling waste anaesthetic gases. To further narrow how the exposure-related effects might be related to proximity to/repetitive exposures to WAG, effects on various types of medical professionals (according to theatre role) including surgeons, surgical assistants (SA), anaesthetists, anaesthetic assistants (AA), nurses and janitors, were evaluated.

## Subjects and methods

### Study population

The study obtained data from a number of hospitals in the Qassim region in Saudi Arabia. A comparative cross-sectional protocol was used. In total, 180 male participants were recruited to the study. Sixty of these were healthy controls (G1) recruited from pharmacy and medical departments, who had not been exposed to WAG. The exposure group was made up of 120 age-matched staff who work in operating rooms fitted with scavenging systems, who had the potential of long-term exposures to WAG. The exposure group was further divided into six subgroups: surgeon (G2), surgical assistants [SA] (G3), anaesthesiologists (G4), anaesthesiology assistants [AA] (G5), nurses (G6) and janitors (G7). The exposure encompasses all worked in operating rooms. Data collected included participants’ medical histories, smoking status, body mass index [BMI determined as weight (kg)/height (m^2^)] and occupational histories (type of anaesthetic gases most commonly used, working times, work shifts and work environ). All participants gave their informed consent before data were collected. Exclusion criteria were applied; thus, participants who were current smokers or had been employed at the hospital for less than one year were excluded. Other criteria for exclusion was ongoing health including immune diseases, such as atopy, for which the participant was receiving treatment or had taken any immune system modulating medication within the previous 6 months. Participants who took antioxidant supplements were also rejected from the study.

Ethics approval for this study was sought and granted by the Research Ethics Committee at the Ministry of Health for Saudi Arabia and the college Ethics committee.

### Collection of blood samples

A 10-ml venous sample of blood was drawn from the participants’ hands between 8:00 and 9:00 A.M after overnight fasting after the work shift. One half of the sample was collected into a silicone-coated tube and centrifuged at 1000×*g* at 4 °C for 15 min to obtain serum. While the other half was transferred to EDTA-coated tubes and processed to obtain plasma. All samples were stored at − 20 °C until require for analysis.

### Plasma fluoride levels

Using the protocols described by Amaral et al. (2018), the plasma fluoride levels were calculated. Briefly, samples were mixed with equal quantities of total ionic strength adjustment buffer (TISAB II); these were homogenised using magnetic stirrers. An Orion 901 ion analyser (Thermo Fisher Scientific, Chelmsford, MA) fitted with an ion-selective and combination pH electrode was used to measure the level of fluoride in the samples. The plasma levels of fluorine were extrapolated from standard curves for fluoride that were generated in parallel. Over the range of 1–48 µM of fluorine, the assay was linear, with correlation coefficients equal to 0.999. The minimum amount of fluoride detectable was 1 µM. Assays were repeated twice for each sample.

### Enzymatic and non-enzymatic antioxidant assays

Malondialdehyde (MDA) was estimated according to the modified method of Olszewska-Słonina et al (2011). The absorbance of supernatant was determined at 530 nm. Total thiobarbituric acid-reactive substances were expressed as MDA, using a molar extinction coefficient for MDA of 1.56 × 105 cm^−1^ M^−1^. To assess glutathione peroxidase (GSHpx) activity, we followed the method characterised by Elgharabawy et al (2018). Activity was determined spectrophotometrically by coupling the oxidation of glutathione and NADPH using glutathione reductase. The decline in NADPH absorbance detected at 340 nm during the oxidation of NADPH to NADP + is marker for GSHpx activity. SOD activity was detected using the method described by Kong et al. (2012). This method depends on the generation of superoxide radicals in the presence of xanthine oxidase. Superoxide radicals react with INT (2-(4-iodophenyl)-3- (4-nitrophenol)-5-phenyltetrazolium chloride) forming formazan dye. The sample absorbance was then monitored at 505 nm for 3 min at 37 °C in a spectrophotometer. Hadwan’s protocol (2018) was followed to evaluated CAT activity. CAT activity detection is based on the monitoring of the rate constant (s^−1^; k) for the decomposition of hydrogen peroxide. The absorbance is performed at 240 nm for 3 min (at 37 °C) in a spectrophotometer. All samples were assayed twice.

### Cytokine assay

An ELISA kit (U-CyTech Biosciences; The Netherlands) was used to measure the concentration of IL-2, IL-4 and IFN-γ cytokines in serum. To each well of a 96-well ELISA plate, diluted capture antibody for the different cytokines were added then incubated at 4 °C overnight. Phosphate-buffered saline with TWEEN-20 was used to wash the plates blocking buffer was added; plates were then incubated at 37 °C for 1 h. The following were added stepwise (1) a serum and cytokine stabilising buffer mixture, (2) biotinylated detector antibody and (3) horseradish peroxidase-conjugated streptavidin polymer. Between each step, the plates were incubated at 37 °C for 1–2 h. The 3,3′,5,5′-Tetramethylbenzidine substrate solution was added and after 30 min, 2 M of sulfuric acid was added to stop the reaction. An ELISA reader was used to get the OD values. Cytokine standards were prepared and their concentrations (pg/ml) established using the standard curve. All samples were measured twice; both inter- and intra-assay coefficients of variation were less than 5% (Corstjens et al. 2011; Hall et al. 2015).

### Statistical analysis

The data were analysed using SPSS v.21.0 (SPSS, Inc., Chicago, IL) software. The results are presented as means ± SEM. To identify whether the differences between the sub-groups (G2–G7) and controls were significant, an one-way analysis of variance (ANOVA) was used to discern significant differences among the exposed subgroups. To compare between the various sub-groups, ANOVA and a Dunnett comparison test were performed (McHugh 2011). The threshold of significance was set at *p* < 0.05. To identify possible relationships between the levels of fluoride (anaesthetic metabolite) in blood and the levels of immune parameters and oxidative stress, Pearson correlation coefficients were used. Correlation analysis was performed using GraphPad Prism version 8.00 for Windows, GraphPad Software, San Diego, USA (Skinner 2018).

## Results

Table 1 presents the socio-demographic characteristics of the participants, all of whom were married males; the characteristics of control (G1) and exposed groups (G2–G7) were matched. The age, BMI and number of working years did not significantly differ between the groups.

**Table 1:** Socio-demographic characteristics and occupational history for control and WAGs exposed study groups

Items	G1 (control)	G2	G3	G4	G5	G6	G7
Age (years)	33.5 ± 1.2	34.7 ± 0.99	34.8 ± 2.1	33.5 ± 0.96	35.0 ± 1.09	35.0 ± 0.98	33.9 ± 1.05
Gender							
Male	60 (100%)	20 (100%)	20 (100%)	20 (100%)	20 (100%)	20 (100%)	20 (100%)
Female	0	0	0	0	0	0	0
Marital status							
Single	0	0	0	0	0	0	0
Married	60 (100%)	20 (100%)	20 (100%)	20 (100%)	20 (100%)	20 (100%)	20 (100%)
Divorcee	0	0	0	0	0	0	0
Separated	0	0	0	0	0	0	0
Body mass index (kg/m^2^)	22.72 ± 0.19	22.70 ± 0.20	22.46 ± 0.20	22.56 ± 0.22	22.58 ± 0.21	22.42 ± 0.24	22.44 ± 0.18
Period of exposure (years)	0	7.5 ± 0.36	6.9 ± 0.45	7.7 ± 0.42	7.1 ± 0.54	7.5 ± 0.31	7.0 ± 0.56

Values expressed as means ± SEM. An one-way ANOVA/Dunnett test. Value significantly different from control at *p ≤ 0.05

*G1* control group, *G2* surgeon-assistant group, *G3* surgeon group, *G4* anaesthesiologist group, *G5* anaesthesiologist-assistant group, *G6* nurses group, *G7* workers group

### Plasma fluoride levels

**Fig. 1:**
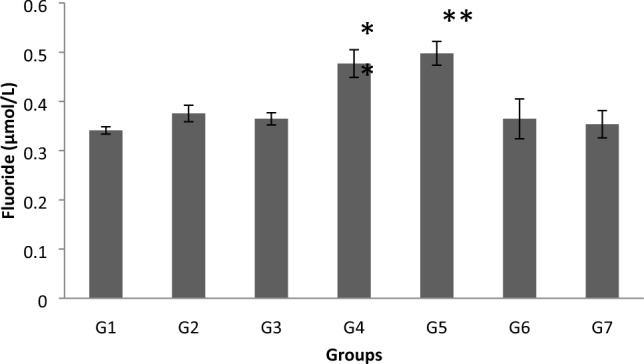
Changes in plasma fluoride concentration in control and waste anaesthetic gases exposed groups. *G1* Control group, *G2* Surgeon-assistant group, *G3* Surgeon group, *G4* Anaesthesia specialist group, *G5* Anaesthesia-assistant group, *G6* Nurses group, and *G7* Workers group. The significance of difference was analysed by one-way ANOVA and Dunnett test (compare all vs. control group) using computer program. Values are expressed as means ± SEM. An one-way ANOVA was significant at *p* < 0.05. Dunnett test was significant from the corresponding control group value at *p* < 0.05, **p < 0.01, ***p < 0.001 and ****p < 0.0001

Compared to controls, the plasma levels of fluoride were elevated in groups G2–G7 (Fig. 1). However, only anaesthesiologists (G4) and their assistants (G5) exhibited significant levels (G4 = 0.44 ± 0.02 and G5 = 0.48 ± 0.01, vs. G1 = 0.35 ± 0.01). The plasma levels of fluoride in G4 and G5 were significantly greater than the levels detected in the other exposure groups (G2, G3, G6 and G7).

### Changes in plasma oxidative stress markers

**Fig. 2:**
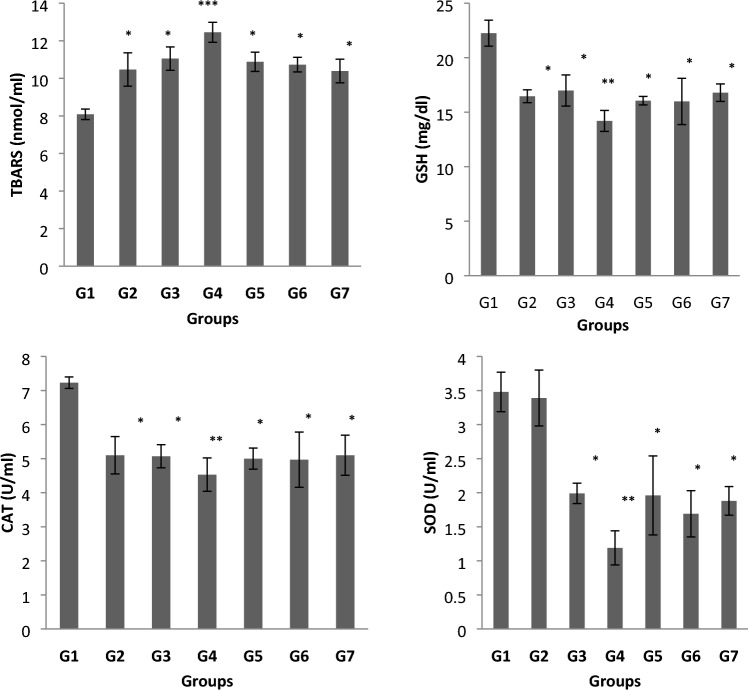
Changes in plasma thiobarbituric acid reactive substances (TBARS), catalase, Glutathione (GSH) and superoxide dismutase (SOD) levels in control and waste anaesthetic gases exposed groups. *G1* Control group, *G2* Surgeon-assistant group, *G3* Surgeon group, *G4* Anaesthesia specialist group, *G5* Anaesthesia-assistant group, *G6* Nurses group, and *G7* Workers group. The significance of difference was analysed by one-way ANOVA and Dunnett test (compare all vs. control group) using computer program. Values are expressed as means ± SEM. one-way ANOVA was significant at *p* < 0.05. Dunnett test was significant from the corresponding control group value at *p < 0.05, **p < 0.01, ***p < 0.001 and ****p < 0.0001

Compared to the control group, the plasma levels of TBARS were significantly higher in the exposed group (Fig. 2). The plasma levels of TBARS in the anaesthesiologist group were significantly greater than in any of the other exposure sub-groups. However, as Fig. 2 demonstrates, no statistically significant difference in the plasma levels of TBARS was detected between the other exposure sub-groups (G2, G3, G5, G6 and G7).

Figure 2 also demonstrates that plasma levels of CAT, GSH and SOD is significantly lower in groups G2-G7 than G1. Between the exposure groups, the plasma levels of CAT, GSH and SOD were significantly lower in the group of anaesthesiologists (G4) than all the other subgroups. The levels of CAT, GSH and SOD in the surgical assistant, anaesthesiology assistant, nurses and janitorial groups did not differ significantly from each other.

### Changes in cytokines

**Fig. 3:**
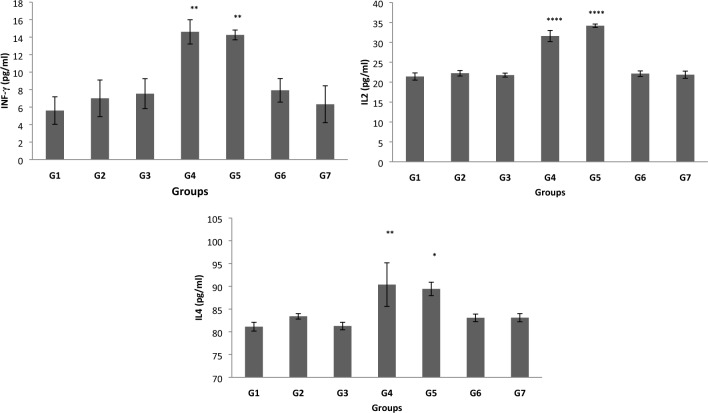
Changes in serum interferon (IFN-γ), interleukin 2 (IL2) and interleukin 4 (IL4) levels in control and waste anaesthetic gases exposed groups. *G1* Control group, *G2* Surgeon-assistant group, *G3* Surgeon group, *G4* Anaesthesia specialist group, *G5* Anaesthesia-assistant group, *G6* Nurses group, *G7* Workers group. The significance of difference was analysed by one-way ANOVA and Dunnett test (compare all vs. control group) using computer program. Values are expressed as means ± SEM. one-way ANOVA was significant at *p* < 0.05. Dunnett test was significant from the corresponding control group value at *p < 0.05, **p < 0.01, ***p < 0.001 and ****p < 0.0001

The serum levels of IFN-γ, IL2 and IL4 in all groups are presented in Fig. 3. Compared with the control group, the levels of cytokines were significantly elevated in the anaesthesiologist and anaesthesiology-assistant groups. However, no other statistically significant differences were detected between the other WAG-exposed groups and the control group.

### Pearson correlation coefficient of different studied parameters in different studied groups

**Fig. 4:**
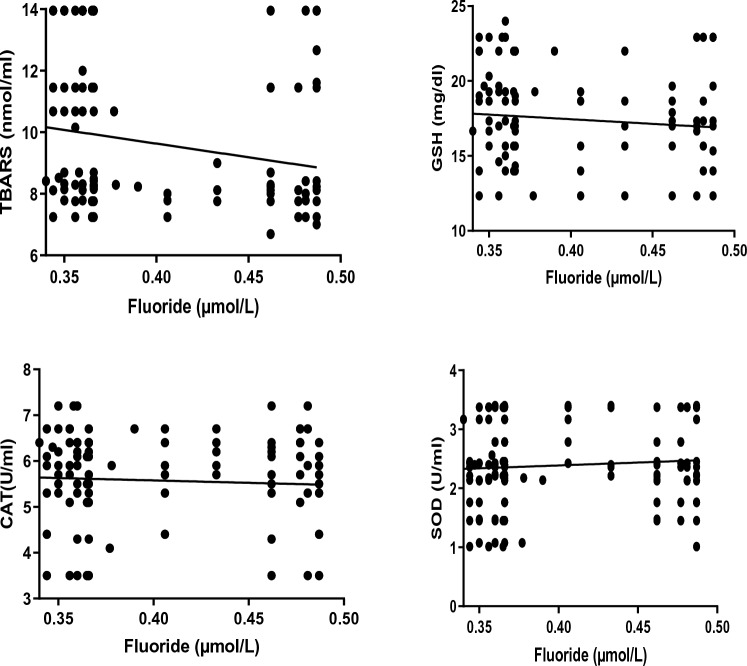
Correlation of plasma thiobarbituric acid reactive substances (TBARS), catalase, glutathione (GSH) and superoxide dismutase (SOD) with plasma fluoride levels in waste anaesthetic gases exposed groups

**Fig. 5:**
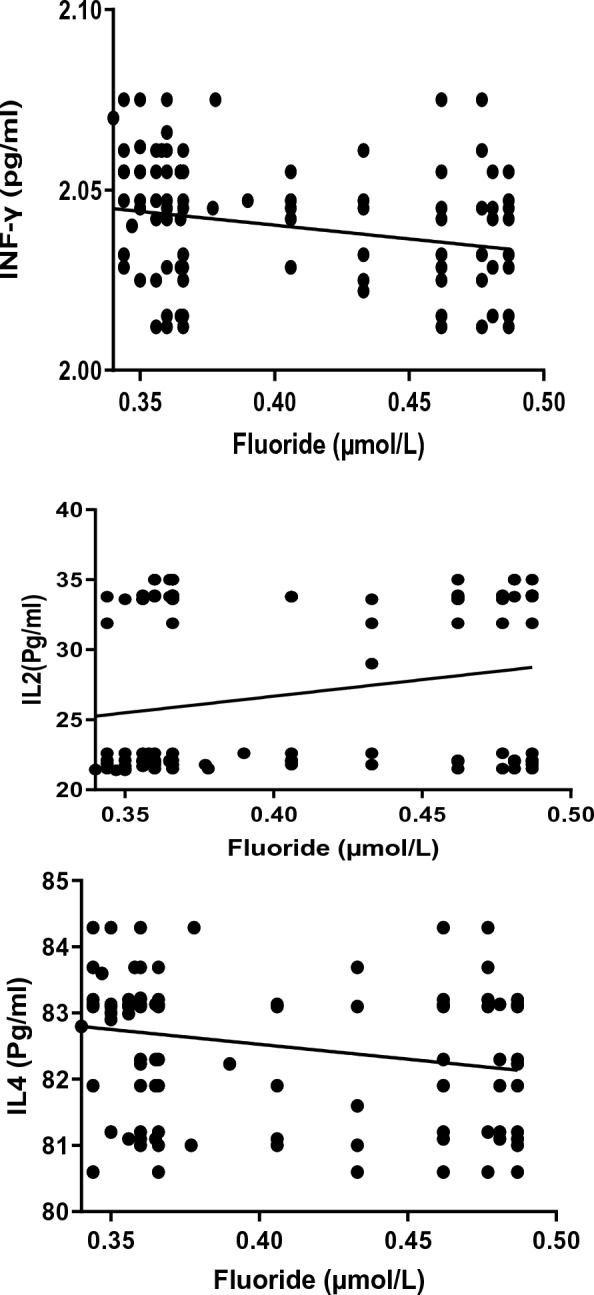
Correlation coefficient of serum IFN-γ, IL2, IL4 with plasma fluoride levels in waste anaesthetic gases exposed groups

The data in Table 2 and Fig. 4 show that there is a significant negative correlation between plasma CAT, GSH, SOD and TBARS and total fluoride. Yet in different groups, a significant positive correlation was identified between the plasma levels of TBARS with CAT, GSH and SOD. Table 3 and Fig. 5 present the correlation coefficient (*r*) for the relationship between IFN-γ, IL2 and IL4 and fluoride in the different groups. Fluoride correlates negatively and significantly with IFN-γ and positively and significantly with IL2. No significant relationship was identified between fluoride and IL4 (Table 3 and Fig. 5).

**Table 2:** Correlation coefficient (*r*) of SOD, CAT, GSH and with fluoride and TBARS for control and WAGs exposed study groups

Parameter	SOD	CAT	GSH	TBARS
Fluoride	− 0.4765*	− 0.5035*	− 0.4891*	− 0.6542**
TBARS	0.6753**	0.7816***	0.4984*	–

*TBARS* thiobarbituric acid reactive substances, *CAT* catalase, *GSH* glutathione, *SOD* superoxide dismutase levels

***Correlation is significant at the 0.001 level (2-tailed)

**Correlation is significant at the 0.01 level (2-tailed)

*Correlation is significant at the 0.05 level (2-tailed)

**Table 3:** Correlation coefficient (*r*) of INF-γ, IL2 and IL4 with fluoride for control and WAGs exposed study groups

Parameter	INF-γ	IL2	IL4
Fluoride	− 0.8135****	0.9242****	− 0.3884

*IL2* interleukin-2, *IL4* interleukin-4, *INF-γ* interferon gamma levels

***Correlation is significant at the 0.001 level (2-tailed)

**Correlation is significant at the 0.01 level (2-tailed)

*Correlation is significant at the 0.05 level (2-tailed)

## Discussion

Hospital staff who work in the operating room are potentially exposed to waste anaesthetic gas, which can pose risks to their health. Exposure to WAGs increases the potential of developing haematological, hepatic immunological, neurological, renal and reproductive system diseases. However, this claim is contentious and according to Dittmar et al. (2015), the level of hazard posed to the health of operating room staff from chronic exposure to WAGs is debatable.

This study examined factors in staff working in operating rooms in hospitals in the Qassim region. Sevoflurane, desflurane and isoflurane, respectively, have 7, 6 and 5 fluoride ions; they are metabolised to produce inorganic fluoride (Lin et al. 2013). During short surgery and longer ICU duration of using anaesthetic, there is a rise in the serum levels of fluoride. Given its greater fluoride content and metabolism, serum fluoride levels are higher after administering sevoflurane than isoflurane (Perbet et al. 2014). This study observed greater plasma levels of fluoride in all exposed groups (G2–G7) than in the control group. Furthermore, the level was higher in the anaesthesiologists and their assistants than in the other groups of operating room staff. Indeed, the plasma levels of fluoride were comparable in all the other WAG-exposed groups.

A number of drugs and diseases cause oxidative damage, and there are similarities between the manifestations of oxidative damage, regardless of cause (García-Sánchez et al. 2020). Oxidative stress is the product of oxygen-containing free radicals, known as reactive oxygen species (ROS), being created faster than they are cleared, creating an imbalance (Elgharabawy et al. 2018). Whilst ROS are key to normal cellular function, undertaking essential roles in cell signalling and destroying harmful pathogens, when the levels of ROS are excessive, cellular dysfunction can occur (Reuter et al. 2010; Basuony et al. 2015).

Maintaining balance between ROS creating and elimination is achieved by several enzymatic and non-enzymatic antioxidant systems; these have been the focus of research in relation to their ability to protect against various chronic diseases (Lim 2014). Oxidative stress can be initiated by anaesthetic gases. A study of medical staff exposed to WAGs found the staff exhibited basal DNA damage and elevated IL-17A (Braz et al. 2020). The same study also observed these staff had a slightly higher incidence of micronucleus but there no significant change to their levels of oxidative stress biomarkers. However, patients who have undergone cholecystectomy, hysterectomy, thoracotomy abdominal and orthopaedic surgeries exhibit DNA damage, inflammation and elevated oxidative stress (Lee et al. 2015). According to Türkan et al. (2011), the level of malondialdehyde in lungs and activity of anti-oxidative enzyme was increased by sevoflurane. The aware of the risks posed by WAGs, efforts to improve operating room environments so as to minimise staff exposure to WAG, total elimination of WAG-contaminated air is unachievable (Asefzadeh et al. 2012). The presence of a potential relationship between oxidative stress and occupational exposure to WAGs is an under-researched topic (Lee et al. 2015). However, elevated numbers of DNA breaks and reduced antioxidant and enzyme capacity were identified in nurses working in operating rooms that lacked a scavenging system. The nurses had an average of 14.5 years of exposure, primarily to desflurane, isoflurane, sevoflurane and nitrous oxide (Izdes et al. 2010). ROS and reactive metabolites can be produced by halogenated anaesthetics. Nitrous oxide is associated with lower levels of cyanocobalamin, which promotes the development of hydroxyl radicals/ROS and superoxide (Costa Paes et al. 2014).

This study found that compared to controls, operating room staff had significantly elevated plasma TBARS, a marker of ROS concentration, indicating lipid peroxidation and significantly reduced plasma levels of CAT, GSH and SOD. Also, compared to the other WAGs exposed groups, the plasma levels of TBARS were statistically significantly greater, and the plasma levels of CAT, GSH and SOD were significantly reduced. However, our findings contradict those of Malekirad et al. (2005), who found that operating room staff exposed to halothane and N_2_O for nine years experienced a reduction in antioxidant thiol groups and a significant increase in lipid peroxidation due to thiobarbituric acid-reactive compounds. Yet, Türkan et al. (2005) reported that plasma GPX and SOD antioxidant enzymes were reduced in staff in Turkish operating rooms with partial scavenging systems exposed to desflurane, enflurane, halothane, isoflurane and sevoflurane. Support for our results also come from Costa Paes et al. (2014), who reported damaged antioxidant states and DNA in medical residents with occupational exposure to WAGs. The results of the current study are consistent with those of Lucio et al. (2018); they noted that operating room staff exposed to anaesthetic gases were more vulnerable to genotoxic and oxidative stress effects. These various results highlight the importance of operating rooms to have appropriate systems in place to eliminate as far as possible the staff’s exposure to noxious working environments. Anaesthesia can exert direct influence upon the production of ROS by disrupting the anti-oxidant system, which in turn can reduce hepatic blood flow (Doğru et al. 2017).

The present investigation and other studies are indicative of oxidative stress being increased in operating room staff exposed to WAGs due to suppression of protective mechanisms. An association is made between the creation of the superoxide and hydrogen peroxide and anaesthetic gases and vapours; these free radicals are liable to cause lipid peroxidation.

ROS is neutralised by direct interaction with GSH, thus GSH is used by enzymes, such as glutathione reductase and glutathione peroxidase, to eliminate ROS. Our study noted a reduction in GSH, which could account for the observed increase in lipid peroxidation. Türkan et al. (2011) reports that sevoflurane exerts an antioxidant effect as evidenced by erythrocyte levels of antioxidant enzymes and MDA in abdominal surgery patients. On the other hand, Orosz et al. (2014), assert that sevoflurane (1.9%) is safe to use, as there is no evidence that it causes DNA damage or affects the levels of lipid peroxidation.

To protect itself from various pathogens, the immune system has evolved diverse and adaptive defensive mechanisms that act rapidly and precisely against the invader. Immune responses that are inadequate or slow to effect are less protective, potentially leading to prolonged disease states; conversely, extreme or poorly regulated immune responses can give rise to autoimmune diseases. There is a robust relationship between inflammation and oxidative stress, each able to initiate the other (Costa Paes et al. 2014; Chaoul et al. 2015). Cytokines are soluble mediators that are biosynthesised by inflammatory cells; these proteins release ROS to recruit other cells to the site of tissue damage, which exacerbates inflammation (Ferguson 2010). Having been activated by antigens, IL-2 is biosynthesised mainly by T-helper cells and, to a lesser extent NK cells; this cytokine is essential fornaïve T cells to grow, proliferate and differentiate into the various effector T cells. Through its autocrine signalling mechanism, IL-2 causes the clonal expansion of antigen-specific T cells and promotes their synthesis of IFN-γ and tumour necrosis factor-α (TNF-α). The IL-2 receptor (IL-2R), which is the means by which IL-2 initiate signals, is made up of three subunits: an alpha unit that is unique to IL-2(IL-2Ra), a β subunit that is also expressed by IL-15 and a γ subunit that is found in IL-4, IL-7, IL-9 and IL-15 (Malek and Castro 2010). Cell responses mediated by TH1 are related to the function of IFN-γ, TNF-α, IL-2 and IL-12; the effect is to promote cellular cytotoxic immunity (Annunziato et al. 2015). Meanwhile, humoral immunity is associated with TH2-mediated responses and is linked to IL-4, IL-5 and IL-13 function. The serum levels of IFN-γ, IL2 and IL4 were higher in the operating room staff included in this study than the levels in controls. Furthermore, among anaesthesiologists and their assistants, the serum levels of IFN-γ and IL-2 were elevated compared to the other operating room staff. However, anaesthesiologists had uniquely elevated serum levels of IL-4, exceeding those of all the other sub-groups of operating room staff exposed to WAGS. According to Stollings et al. (2016), significant increases in the levels of cytokine have been identified in a number of studies examining the effects of exposing patients to levels halogenated anaesthetics. Several pulmonary functions were adversely affected in lung cancer patients undergoing resection due to the secretion release of inflammatory factors following single-lung ventilation with 6–8% sevoflurane (Jin et al. 2013). Although Chaoul et al. (2015) observed significant increases in the levels of the pro-inflammatory cytokine, IL-8, in staff occupationally exposed to anaesthetic gases; the same staff exhibited a slight reduction in IL-10. However, the researchers did not identify any significant difference in the plasma levels of IL-1b, IL-6, IL-12 and TNF-α.

The present study was limited by the strict inclusion criteria that limited the sample size and excluded females, which may reveal other data. A larger, more heterogeneous sample may provide new insights and facilitate a deeper understanding of the effects of WAGs upon oxidative stress and cytokine in operating room staff.

The present study leads us to conclude that changes to serum levels of cytokines and oxidative stress arise in operating room staff as a consequence of exposure to WAGs. Thus, these staff are vulnerable to experiencing immunotoxicity. To raise awareness and help address the situation, programmes should be developed that aim to educate healthcare staff to reduce their risks of immunotoxicity by managing these gases. Furthermore, safety protocols for using anaesthesia should be re-assessed frequently and improvements be implemented where possible. Further research that builds on the findings presented here is required to investigate in more detail the health effects of occupational exposure to WAGs.
