# Correction to: Effect of inhaled anaesthetics gases on cytokines and oxidative stress alterations for the staff health status in hospitals

**DOI:** 10.1007/s00420-024-02052-4

**Published:** 2024-02-07

**Authors:** Khaled A. AL-Rasheedi, Abdulmajeed A. Alqasoumi, Ashraf M. Emara

**Affiliations:** 1 Khyber General Hospital, Ministry of Health, Khyber, Saudi Arabia; 2 Department of Pharmacy Practice, College of Pharmacy, Qassim University, Qassim, Saudi Arabia; 3 Department of Pharmacology and Toxicology, College of Pharmacy, Qassim University, Buraidah, Qassim Saudi Arabia; 4 Department of Clinical Toxicology, Faculty of Medicine, Tanta University, Tanta, Egypt

**Correction to: International Archives of Occupational and Environmental Health (2021) 94:1953–1962** 10.1007/s00420-021-01705-y

In this article the statement in the Funding information section is included after the publication which is given below.

“The researchers would like to thank the Deanship of Scientific Research, Qassim University for funding the publication of this project’’.

The original article has been corrected.

